# Combined Puestow and choledocoduodenostomy for concomitant large pancreatic duct and primary choledochal stones: A case series

**DOI:** 10.1016/j.ijscr.2018.10.051

**Published:** 2018-11-12

**Authors:** Adeodatus Yuda Handaya, Nova Yuli Prasetyo Budi, Aditya Rifqi Fauzi

**Affiliations:** a Digestive Surgery Division, Department of Surgery, Dr. Sardjito Hospital/Faculty of Medicine, Public Health, and Nursing, Universitas Gadjah Mada/Yogyakarta, Indonesia; b Faculty of Medicine, Public Health, and Nursing, Universitas Gadjah Mada, Yogyakarta, Indonesia

**Keywords:** Pancreatic duct stones, Choledochal stones, Pancreatojejunostomy Roux- en-Y, Choledoco-duodenostomy

## Abstract

**Introduction:**

Multiple and large pancreatic duct stones concomitant with primary choledochal stones is a rare case. Patients usually present with recurrent jaundice and signs of pancreatitis. Endoscopic retrograde cholangiopancreatography (ERCP) is the leading method to manage the patients. But ERCP has difficulties when facing the multiple and large stones

**Presentation of case:**

Our first case was a 51-years-old man who was admitted to our surgery unit with a diagnosis of chronic pancreatitis. Plain abdominal radiogram, Abdominal MSCT and Magnetic Resonance Cholangiopancreatography (MRCP) showed opacity suspected as stone at the pancreatic duct and distal part of the common bile duct. The second case was a 48-years-old female with the clinical presentation of left upper quadrant pain and history of chronic pancreatitis and intermittent jaundice. Plain abdominal radiogram and MRCP revealed multiple stones in the main pancreatic duct and common bile duct stones. Our third case was female, 60-years-old, who was hospitalized with jaundice and recurrent upper abdominal pain with a history of open cholecystectomy one month previously. Radiologic examination showed multiple stones in the main pancreatic duct and common bile duct. Combined longitudinal pancreatojejunostomy Roux-en-Y and Choledoco-duodenostomy were performed successfully in all cases. Postoperative follow-up showed good recovery of all patients.

**Discussion:**

Since ERCP is not proper to be used for multiple and large pancreatic duct stones, we performed a combination of longitudinal pancreatojejunostomy Roux-en-Y and choledoco-duodenostomy to treat the patients and prevent the recurrence.

**Conclusion:**

The incidence of multiple pancreatic duct stones and large choledochal stones is infrequent. Surgical treatment with combined longitudinal pancreatojejunostomy Roux-en-Y and Choledoco-duodenostomy is safe and effective to resolve jaundice and recurrent pain caused by chronic pancreatitis.

## Highlights

• An alternative for endoscopy retrograde cholangiopancreatography (ERCP).
• Minimal intraoperative bleeding.
• This helps reduced length-of-stay in hospital.
• This procedure can be an alternative to prevent recurrence.

## 1 Introduction

Pancreatic duct stones refer to calcification in the pancreatic duct. It is categorized into pancreatic duct stone (real stone) and pancreatic parenchyma calcification (false stone). Pancreatic duct stone concomitant with choledochal stone is a rare disease. Pancreatic ductal stones are often associated with chronic pancreatitis. The most common cause of chronic pancreatitis in the UK is alcohol, and other causes are hereditary or idiopathic. Choledochal stone is a stone found in the common bile duct, due to precipitation of the bile caused by an imbalance between bile acids, cholesterol, and lecithin, or secondarily from the migration of gallbladder stones into the common bile duct. Choledochal stone will give symptom like recurrent jaundice and may be associated by cholangitis (fever, jaundice, and pain: Charcot’s triad) or even with shock and deterioration of consciousness (Reynold’s Pentad). The plain abdominal radiogram finding suggestive of pancreatic duct stones and choledochal stone is the opacity in the medial and right upper quadrant. CT scan will reveal chronic pancreatitis with the dilation of the pancreatic duct and also calcification of the intrapancreatic duct. MRCP performed will show moderate to severe dilatation of the common bile duct with opacity at the distal part, together with opacity and dilatation of the main pancreatic duct. The pancreatic ductal stone found with choledochal stones generally occur in the late stage of chronic pancreatitis and recurrent jaundice with chronic cholangitis [[1], [2], [3]].

Pancreatic duct stone is tough to diagnose timely due to the absence of specific clinical manifestations. Currently, laboratory tests for pancreatic duct stone have no specific indices. Radiological examinations such as ultrasonography (USG), computed tomography (CT), endoscopic retrograde cholangiopancreatography (ERCP) and magnetic resonance cholangiopancreatography (MRCP) are used to confirm this disease. As the foremost choice for diagnosis of the disease, ultrasonography is an easy and low-budget examination. Management of pancreatic duct and choledochal stone continues to develop, and it is dependent on the available resources. ERCP has become the leading method for both diagnosis and treatment of pancreatic duct stone recently [2]. This method is also more advantageous for non-invasive and repeatable treatment. The success of endoscopic intervention as a less invasive procedure in the treatment of pancreatic stone is partly due to the improvement of endoscopic techniques. However, pancreatic duct stones approximately 5 mm or greater are often not amenable to conventional management with sphincterotomy, stricture dilation, or stone retrieval with basket balloon catheter dilation.

ERCP usually has difficulties due to basket size, residual stones and need a longer duration of surgery. If the stones are located in the body of the pancreas, they can be treated with the procedures consisted of a lateral Roux-en-Y pancreatojejunostomy (Thal procedure). Distal pancreatectomy with splenectomy and end-to-side Roux-en-Y pancreatojejunostomy or also called with DuVal procedure, resection of the spleen and tip of the pancreatic tail with a filleted pancreatic duct and Roux-en-Y pancreatojejunostomy (Puestow procedure), and pancreaticoduodenectomy (Whipple procedure) [4,5]. Successful removal of pancreatic duct stones can reduce pain and improve pancreatic function, and patients will have regressed ductographic changes of chronic pancreatitis and decreased the diameter of the pancreatic duct [2].

The outcomes of endoscopic treatment were reported to be equivalent to those of surgery. In the present study, the indications for the endoscopic procedure were ≤ 3 stones, stones confined in the head and body of the pancreas, absence of restricted pancreatic duct, PDS diameter ≤ 10 mm, and noncompacted stone(s) [8]. Additional and larger stones were retrieved successfully using a modified metallic stent. For patients who need treatment but do not meet the indications above, or for whom conservative therapy fails, surgery is vital [9]. Choledoco-duodenostomy shunt can be performed to prevent recurrent stones formation in the common bile duct.

We conducted a case report study with the subjects are patients from secondary and tertiary hospitals in Yogyakarta, Indonesia. This case series is an operator experience as a digestive surgeon. This research work has been reported in line with the PROCESS criteria [10].

## 2 Cases reports

### 2.1 Case 1

A 51-year-old man was admitted to our surgical unit with a diagnosis of chronic pancreatitis. He was suffering from episodes of continuous abdominal pain for the last one year. The pain was radiated from the right upper quadrant of the abdomen to the back and was associated with nausea, partially relieved by injectable analgesics and aggravated by food ingestion. This challenge led to the loss of appetite then to weight loss which also worsened by the presence of occasional episodes of malabsorption and the development of insulin dependent diabetes mellitus. There was no family history of the similar condition in parents, siblings or first-degree relatives.

Moreover, there was no history of abdominal trauma in the past. The patient had a history of admissions to different hospitals for the recurrent pain attacks. However, apart from this illness, he had never been to hospitals for any other medical or surgical condition. He was on analgesics, insulin therapy, and pancreatic enzyme supplementation and had never been allergic to the exposed medications.

The physical examination showed an emaciated man looking older than his chronological age, appeared pallor but no clinical evidence of jaundice. Abdominal examination was normal. Chest examination found no remarkable findings, and the rest of the physical examination was normal. The laboratory examination showed an increased level of blood sugar, normal levels of serum amylase, renal function tests and liver function test (LFT). No attempt was made to establish the insufficiency of the exocrine pancreatic function. Chest X-Ray and ECG were normal.

**Fig. 1:**
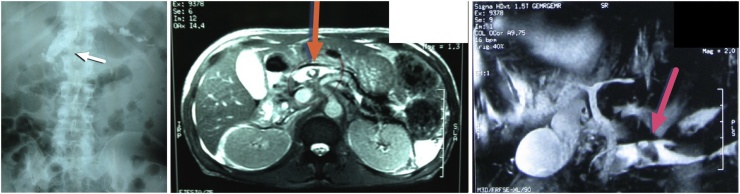
Imaging showing opacities: a. Plain abdominal X-ray in the region of middle and right upper quadrant abdomen; b. MSCT and c. MRCP is showing opacities in pancreatic duct and distal common bile duct.

Plain radiology showed opacities in the middle and right upper quadrant abdomen (Fig. 1a). CT abdomen was performed to rule out other associated pathologies like pancreatic pseudocyst, pancreatopleural, pancreatogastric or pancreatocolonic fistulae as the surgical procedure would have been different in the presence of any of the complications. Contrast-enhanced abdominal CT scan revealed impacted stones at the ampulla within the distal bile duct and pancreatic duct. The remaining parts of the pancreas showed normal result with no associated features of chronic pancreatitis (Fig. 1b). Since the contrast-enhanced abdominal CT scan indicated impacted stones in the distal bile duct at the ampulla, MRCP was performed. Initial MRCP suggested stones within the main pancreatic duct (MPD) in the head of the pancreas and dilated common bile duct suggested stone in the distal part of it (Fig. 1c).

### 2.2 Case 2

A female patient age 48-years-old was admitted with a diagnosis of obstructive jaundice with chronic pancreatitis. She was suffering recurrent abdominal pain in the back for the last eight months and recurrent jaundice in the last three months and diagnosed with hepatitis at a district hospital before referred to our hospital.

Physical examination found slight jaundice at the sclera and skin, with abdomen within normal limit. Chest examination found no remarkable findings. Laboratory results showed raised the level of blood sugar, but we found normal values of serum amylase, renal function tests and Liver Function Tests (LFT’s). Chest X-Ray and ECG were normal.

**Fig. 2:**
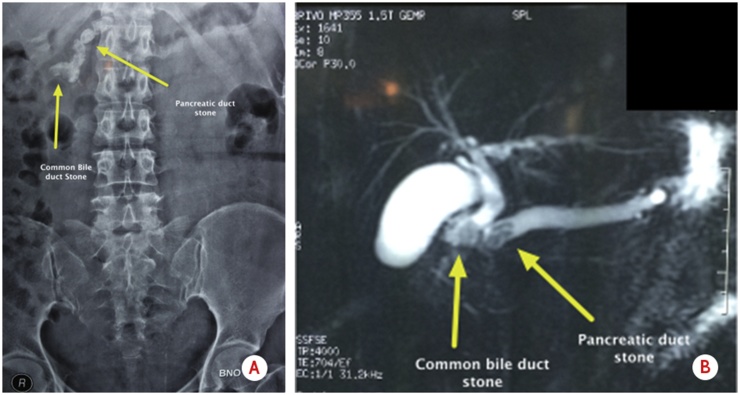
Imaging: a. Plain radiogram showing opacities and areas dilatation in the region of the distal bile duct dan pancreatic duct; b. MRCP showed radiolucent stones in the distal of the common bile duct and the pancreatic duct.

Plain radiogram (Fig. 2a) showed opacities and areas of dilatation in the region of the biliary tract and pancreatic duct. MRCP was performed, and the result suggested a radiolucent stone in the distal of the common bile duct and the duct of the pancreas (Fig. 2b).

### 2.3 Case 3

Female, 60-year-old, was hospitalized with recurrent upper abdominal pain. The patient complained about right upper quadrant pain and recurrent yellowish of her eyes. One month previously, she underwent open cholecystectomy due to cholelithiasis. Physical examination revealed jaundice of the skin and sclerae and tenderness in her epigastric region. Laboratory tests showed total bilirubin 7.05 mg/dL, direct bilirubin 5.62 mg/dL, Gamma GT 475 U/L, and Alkaline Phosphatase 511 U/L. Other laboratory values were within normal limit.

**Fig. 3:**
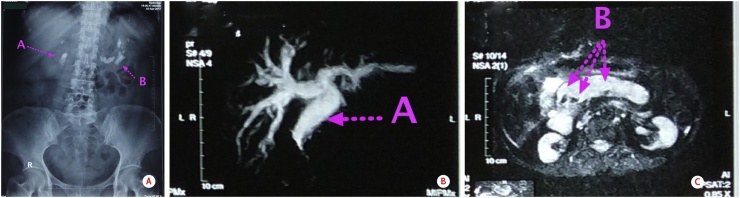
a. Abdominal X-ray; b and c. MRCP showed common bile duct and pancreatic duct dilatation and stone (Arrow A: Stone in the common bile duct, Arrow B: Stones in the pancreatic duct).

On her plain abdominal radiogram, there were opacities in the abdomen region suspected as stone in common bile duct and pancreatic duct (Fig. 3a). MRCP revealed opacities suggested as stones in the pancreatic duct and distal of the common bile duct (Fig. 3b and c).

## 3 Therapeutic intervention - surgery

For every patient with jaundice, we give vitamin K injection. Coagulation test was performed. Pre-operation counseling was done, and informed-consent was taken after discussing the early and late post-operation morbidity and mortality along with the benefits of the procedure. Because the stones were large (> 5 mm) and multiple, we planned to perform open exploration of the common bile duct and pancreatic duct.

**Fig. 4:**
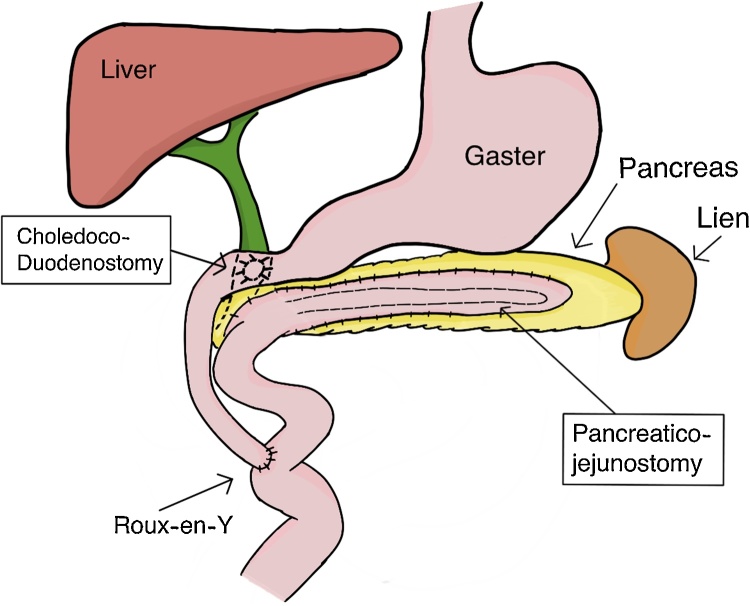
Combined longitudinal pancreatojejunostomy Roux-en-Y and choledoco-duodenostomy procedure.

Under G/A left subcostal incision was done. By opening the gastrocolic ligament, this will expose an area of the pancreas. Combined longitudinal pancreatojejunostomy Roux-en-Y and Choledoco-duodenostomy procedure were performed in all of the patients (Fig. 4) with silk 3/0. We chose to combine these procedures to prevent the recurrent formation of the stones in the common bile duct and main pancreatic duct and also to reduce the risks of chronic pancreatitis and cholangitis.

**Fig. 5:**
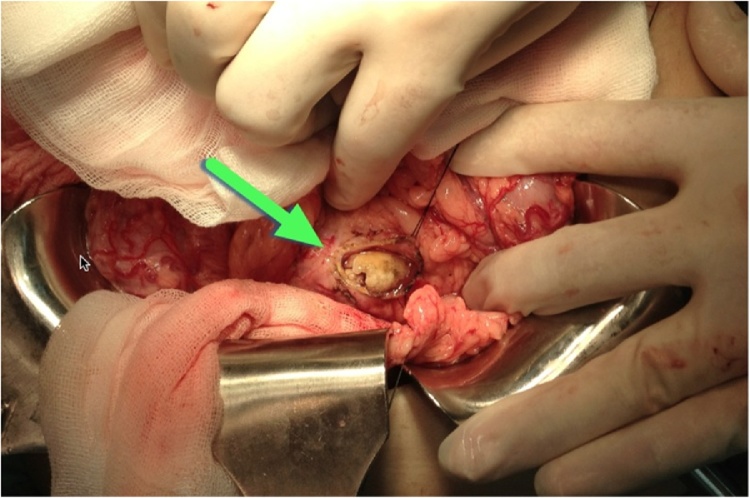
The main pancreatic duct was opened after confirming it by palpating the stone.

**Fig. 6:**
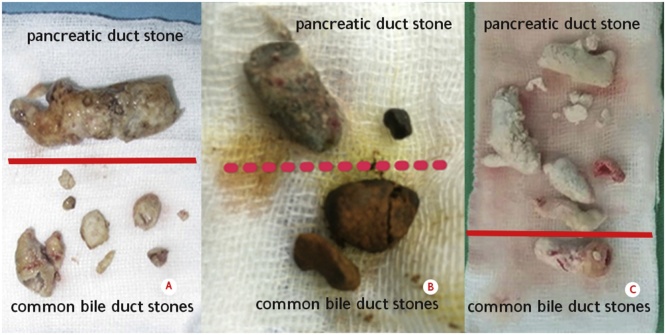
Stones found in the surgery: a. Case 1; b. Case 2; c. Case 3 (A: stones in the common bile duct, B: stones in the pancreatic duct).

The main pancreatic duct was opened after confirming it (Fig. 5) by palpating the stone and aspirating the pancreatic juice. Stone then was removed. We also opened the common bile duct (CBD) to explore the distal part and removing the stones. In case number 3, the surgical techniques were more challenging due to adhesion of the previous cholecystectomy, so firstly we had to performed adhesiolysis. As the results of this surgery, we found multiple stones at the pancreatic duct and common bile duct (Fig. 6).

## 4 Follow-up and outcomes

All patients made an uneventful recovery and have been asymptomatic with no recurrence of pain, jaundice, cholangitis after choledocoduodenostomy shunt (Sump syndrome) nor pancreatitis signs and symptoms.

The authors attest that full and informed consent was obtained from every single patient who has undergone medical treatment in our Hospital. The informed consent form also declared that patient data or samples would be used for educational or research purposes. This work has been registered in the publicly accessible database and having a unique identifying number: researchregistry4378.

## 5 Discussion

Pancreatic duct stones concomitant with stone in the distal common bile duct is a rare finding. Its pathogenesis remains enigmatic and unknown. Pancreatic duct stones are most often associated with chronic pancreatitis. Meanwhile, the stone in the common bile duct might be primary or secondary stones that formed in the gallbladder and migrate into the bile duct. Radiological features of chronic pancreatitis are readily evident in the presence of these stone. However, large size staghorn solitary or multiple stones in the main pancreatic duct are rare. Two primary patterns of calcification are believed to exist: an intraductal pattern, representing true stone and a parenchymal calcific pattern, representing “false stone.” Alcoholism stands out as the leading cause of the chronic calcific pancreatic disease [1].

There are two mechanisms of pathogenesis of stones formation that change of the pancreatic fluid component and pancreatic duct obstruction [1]: Chronic pancreatitis. This condition of inflammation has a close relationship with pancreatic duct stone. The pathological changes of the pancreatic parenchyma in pancreatic duct stone are similar to those in chronic pancreatitis. It is generally accepted that stones formation is combined with inflammation, as our records demonstrate. Chronic pancreatitis, however, is not always combined with pancreatic duct stone. It is an essential factor in stone formation, but they are not causally associated. Different opinions on this aspect have been expressed in the past [2]; Alcohol abuse. Alcohol stimulates the excretion of pancreatic enzymes which destroy the pancreatic alveolus and epithelium of the pancreatic duct. Then the components of the pancreatic fluid turn; protein and calcium concentrations increase so that protein emboli are formed, leading to chronic pancreatitis and pancreatic duct stones. In the past, alcohol abuse was regarded as the most important cause of pancreatic duct stone. They had a positive correlation. Many alcoholics did not have pancreatic duct stones while some that did not drink or drank little had them. Many experimental studies have demonstrated a direct correlation between stone formation and long-term alcohol abuse. However, the process of stone formation and chronic pancreatitis due to alcohol abuse is not discussed [3]; Biliary disease. The pancreatic duct and the common bile duct have a common opening in the duodenal papilla. Obstruction of the lower segment of the bile duct often leads to obstruction of the effluent duct of the pancreas and then can cause bile reflux. Changes in enzymes and pathological changes in the pancreas are the consequences. As a result, pancreatic stones and pancreatitis develop [6,7].

In the present case, CT scan had suggested a stone impacted at the distal of common bile duct or ampulla. However, this was disproved at MRCP which revealed a 6 cm solid stone within the pancreatic head. There was a solitary stone of the pancreatic duct without any calcification within the parenchyma. The patient complained about pain and recurrent jaundice caused by choledochal stones. Despite this large staghorn and multiple solitary stones, the patient had previous episodes of chronic pancreatitis and no history of alcohol abuse with no radiological features of chronic pancreatitis [3].

A clue to the diagnosis of the common bile duct and pancreatic stone by MRCP is the presence of radiolucent areas in the dilated pancreatic duct. By contrast, our patient had a radio-opaque shadow in the distal common bile duct and head of the pancreas on CT and MRCP. This opacity may be single or multiple existing as small concretions or well-developed stones 1 to 2 cm in diameter. We propose that the stones may have originated in the pancreatic duct and common bile duct. Combined longitudinal Roux-en-Y Roux-en-Y and the Choledoco-duodenostomy procedure were performed. Bile and pancreatic duct exploration with stone extraction are a safe procedure. We were not doing a hepatojejunostomy because it will make the flow of bile reflux to the pancreatic duct after we performed Roux-en-Y for pancreatojejunostomy. In case of preventing the increased risk of cholangitis on the pancreatojejunostomy procedure, we have made a shunt with a diameter as big as common bile duct from the common bile duct directly to the duodenum as distal as possible so the bile flow easily passing to duodenum [1,4,5].

## 6 Conclusion

Combination procedure of Longitudinal Pancreatojejunostomy Roux-en-Y and the Choledoco-duodenostomy performed for multiple and large pancreatic and primary choledochal stones (size > 5 mm) is a safe procedure. This procedure is a feasible surgery method with minimal morbidity that can be performed in hospitals which do not have facilities of ERCP nor endoscopic-specialized surgeon.

## Conflict of interest

No potential conflict of interest relevant to this article was reported.

## Funding

The authors declare that this study had no funding resource.

## Ethical approval

The informed consent form was declared that patient data or samples will be used for educational or research purposes. Our institutional review board also do not provide an ethical approval in the form of case report.

## Consent

We have obtained all patient’s consent and had the statement included in the consent section in the manuscript. We also do not include any of the patients name or the institution.

## Author contribution

Adeodatus Yuda Handaya conceived the study. Nova Yuli Prasetyo Budi and Aditya Rifqi Fauzi drafted the manuscript and critically revised the manuscript for important intellectual content. Adeodatus Yuda Handaya, Nova Yuli Prasetyo Budi, and Aditya Rifqi Fauzi facilitated all project-related tasks.

## Registration of research studies

researchregistry4378.

## Guarantor

Adeodatus Yuda Handaya.
